# The use of DNA from archival dried blood spots with the Infinium HumanMethylation450 array

**DOI:** 10.1186/1472-6750-13-23

**Published:** 2013-03-15

**Authors:** JiHoon E Joo, Ee Ming Wong, Laura Baglietto, Chol-Hee Jung, Helen Tsimiklis, Daniel J Park, Nicholas C Wong, Dallas R English, John L Hopper, Gianluca Severi, Graham G Giles, Melissa C Southey

**Affiliations:** 1 Genetic Epidemiology Laboratory, Department of Pathology, The University of Melbourne, Victoria, 3010, Australia; 2 Cancer Epidemiology Centre, Cancer Council Victoria, Victoria, 3053, Australia; 3 Life Sciences Computation Centre, Victorian Life Sciences Computation Initiative Carlton, Victoria, 3010, Australia; 4 Cancer and Disease Epigenetics, Murdoch Childrens Research Institute, Department of Paediatrics, The University of Melbourne, Royal Children’s Hospital, Victoria, 3052, Australia; 5 Centre for Molecular, Environmental, Genetic and Analytic Epidemiology, The University of Melbourne, Victoria, 3010, Australia

**Keywords:** DNA methylation, Epigenetics, Infinium HumanMethylation450, Dried blood spots, Guthrie cards, Genome-wide DNA methylation

## Abstract

**Background:**

Dried blood (Guthrie card) spots provide an efficient way to collect and store blood specimens. DNA from this source has been utilised for a number of molecular analyses including genome-wide association studies, but only few studies have tested the feasibility of using it for epigenetic applications, particularly at a genome-wide level.

**Results:**

In this study, we demonstrate the successful use of DNA isolated from archived dried blood spots for the Infinium HumanMethylation450 Beadchip, along with DNA from matched frozen buffy coats. We obtained high quality and reproducible genome-wide DNA methylation profiles using both sample types. We also report high correlations (r > 0.9907) between DNA obtained from matched dried blood spots and frozen buffy coats, sufficient to distinguish between unrelated individuals.

**Conclusions:**

We, thus, demonstrate that DNA from archived dried blood spots is suitable for genome-wide DNA methylation profiling.

## Background

Dried blood spots, or Guthrie Cards are obtained routinely from newborns in many developed countries to screen for metabolic disorders [1]. These specimens also provide a long-term, cost-effective and convenient alternative to freezing blood [2] and have been used by many large epidemiological studies such as the Melbourne Collaborative Cohort Study [3], the Breast Cancer Family Registry [4] and the IARC Biobank (http://ibb.iarc.fr). Given the size of these studies, the ability to use this source of DNA with current genetic research platforms will enable them to make a considerable contribution to our understanding of the genetics of human disease. The successful use of DNA from dried blood spots (involving whole-genome amplification of the DNA to enhance sensitivity) for downstream genome-wide applications such as microarray-based SNP genotyping has been occasionally reported [5,6].

The significance of DNA methylation marks to human health is an emerging field. For instance, aberrant DNA methylation marks both loci-specific and globally are associated with virtually all cancers [7]. Their significance has led to the development of techniques enabling “epigenome-wide” measures. Commonly employed methods include MeDIP-seq (Methylated DNA ImmunoPrecipitation sequencing) and MBD-seq (Methyl-CpG Binding Domain protein sequencing), which involve the enrichment of methylated DNA using antibodies or methyl-binding domains [8]. These methods rely on an efficient affinity between the molecules and, thus, require a large amount of starting DNA and may be prone to potential biases. The Infinium HumanMethylation450 (HM450) Beadchip array (Illumina), the successor to the HM27, enables the detection of DNA methylation levels at 486,685 CpG dinucleotides across the genome and is one of the most comprehensive microarray methods to date for investigating the “methylome” [9]. Early reports have shown this platform to be highly reproducible [10] and to require a relatively small amount of DNA (as low as 500 ng) making the utility of limited DNA available from dried blood spots feasible. Like many other DNA methylation assays, this platform is based on sodium bisulfite conversion of DNA [11]. Although bisulfite treatment can cause extensive degradation to DNA, a few studies have already demonstrated the successful use of DNA extracted from dried blood spots as well as archived tissue specimens for PCR-based loci-specific detection of methylation [12-14]. Furthermore, Beyan and colleagues recently reported a successful use of dried blood spot DNA for genome-wide DNA methylation detections [15]. However, a systemic comparison between Guthrie DNA and their matched frozen counterpart still remains to be addressed.

In this study, we tested the feasibility of using DNA extracted from dried blood spots for genome-wide methylation profile assessment on the HM450 platform. DNA from five individuals was extracted from three year old archived dried blood spots, bisulfite converted and hybridised onto the HM450 platform. The results from this processing regimen were compared with those derived from DNA extracted from matched frozen buffy coat samples. The quality of data, reproducibility, and correlation between the two sample types were assessed.

## Results and discussion

Although dried blood spots provide a valuable bioresource for research, DNA from this source has been shown to deteriorate with prolonged storage [16]. The potential use of DNA from dried blood spots for genome-wide epigenetic applications has, thus, remained speculative. Here, we have successfully demonstrated the utility of dried blood spot DNA for measuring genome-wide DNA methylation using the Infinium HM450 BeadChip platform. We have also compared these methylation measurements against those of matched DNA extracted from buffy coats from the same individuals (bled at the same time) to investigate the commonality of methylation profiles as influenced by the method of sample storage.

### Quality of the samples and reproducibility of the platform

**Figure 1:**
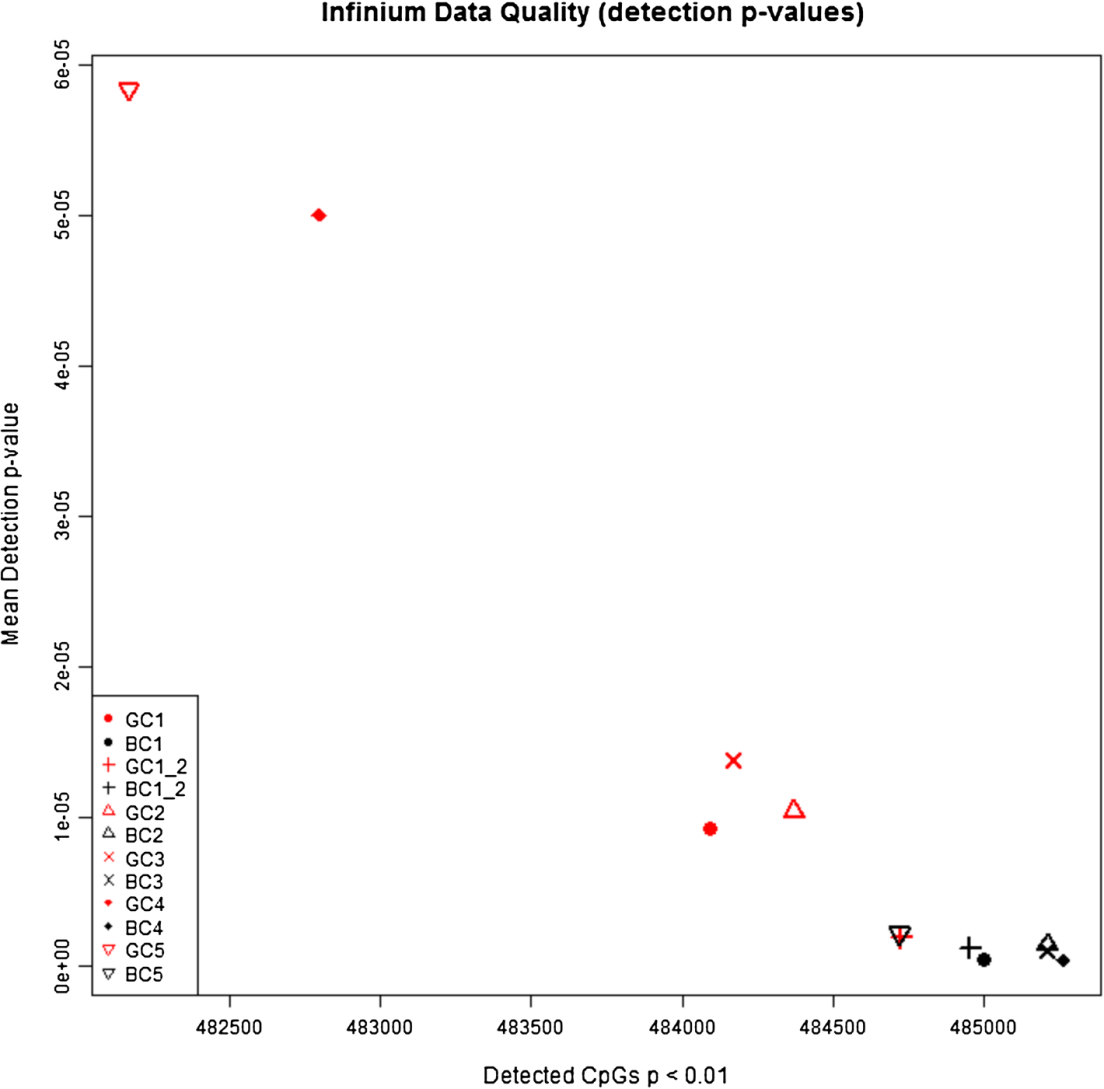
**Mean Detection p-value for each sample is plotted against Number of Detectable CpGs with less than 0.01 Detection p-value.** Dried blood spot samples (GC) are in red and buffy coat samples (BC) are in black.

**Figure 2:**
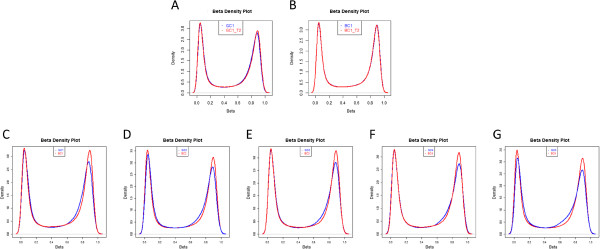
**β-value Density Plots. A**-**B**, Density plots for dried blood spot technical replicates and buffy coats technical replicates. **C**–**G**, density plots for matched samples (dried blood spot (**GC**) versus buffy coat (**BC**)) for each of the five individuals.

No sample analysed exceeded a mean detection p-value of 0.0017 across all probes (Figure 1 and Additional file 1: Table S1). The majority of all individual probes (> 99.8%) for each sample met the commonly accepted quality control detection p-value of 0.01, indicating high quality data were obtained from both sample groups, but the analysis of blood spot DNA generally showed slightly higher mean detection p-values than buffy coat DNA, with fewer probes having a detection p-value below the conventional level of 0.01 (Figure 1). Wilcoxon signed rank test on mean detection p-values revealed significantly higher quality data were obtained from buffy coat DNA than from dried blood spot DNA (p-value = 0.03). This could potentially be due to the poorer quality of DNA obtained from blood spots. After removing probes with higher detection p-values of 0.01 and probes on the X and Y chromosomes, a total of 468,610 analysable probes, common across 12 samples, remained for subsequent analyses. Strong correlations, which is calculated across all probes (r = 0.9961 and 0.9987, Figure 2 and Additional file 2: Figure S1) were observed for β values between 2 pairs of technical replicates, indicating the platform’s highly reproducible data from both dried blood spots and frozen buffy coat DNA, respectively.

### Consistency of DNA methylation between dried blood spot and buffy coat DNA

**Figure 3:**
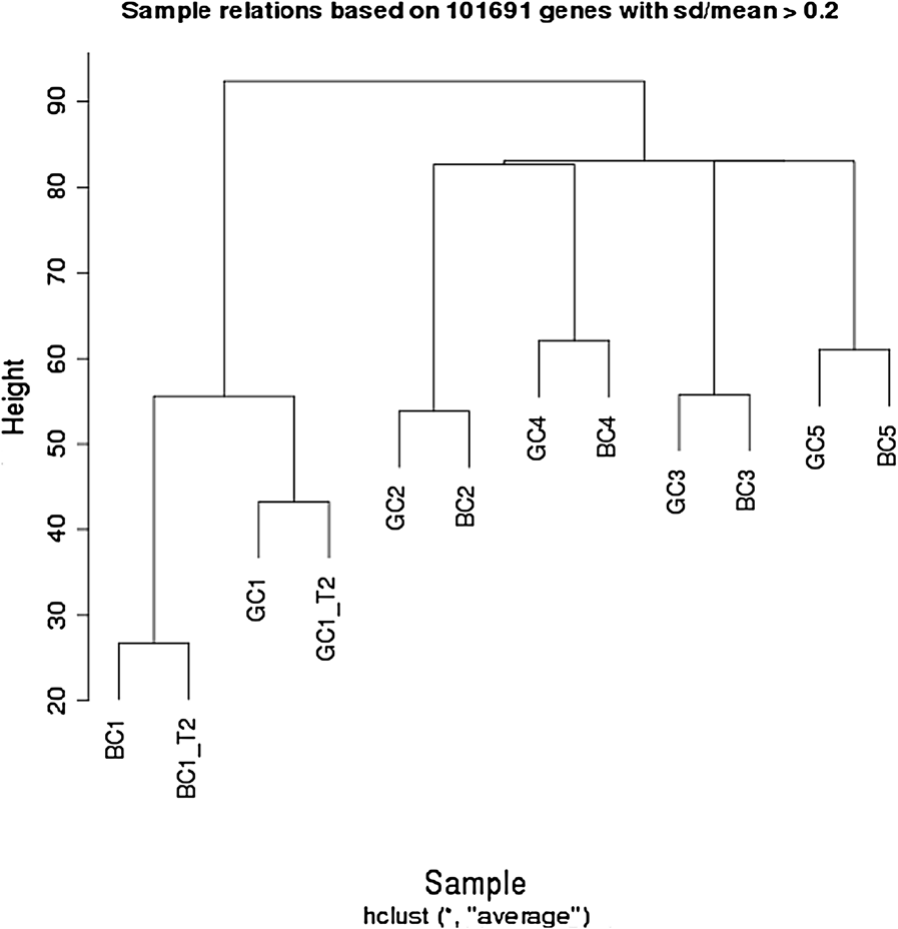
**Unsupervised clustering plot based on the methylation (M) values of the number of variable genes.** Samples from the same individual (GC, dried blood spot; BC, buffy coat) are labelled with the same number. Duplicates from individual 1 are labelled with _T2. The clustering analysis was performed on the top 101,691 most variable probes (CV > 0.2). The variable probes were selected by calculating the Coefficient of Variance (CV, standard deviation/mean).

DNA methylation profiles were compared between dried blood spots and frozen buffy coats collected from the same individuals. Unsupervised clustering analysis of the most variable 101,691 probes (coefficient of variance > 0.2) revealed strong similarities between the two sample types within each individual. DNA methylation differences between individuals were apparent and sufficient to discriminate matched pairs from unrelated samples (Figure 3). When this was repeated on the entire probe set, a similar pattern was detected, where most matched pairs closely clustered (Additional file 2: Figure S1). These effects were also reflected in the Pearson’s correlations (Additional file 2: Figure S2). The means correlation across all probes was higher between technical replicates (r = 0.9932) when compared with matching dried blood spot and buffy coat samples (0.9907). The means correlation was lowest between unrelated individuals (0.9873). These findings suggest that DNA from dried blood spots could be used as a representative alternative to that derived from buffy coats for genome-wide DNA methylation profiling.

Although dried blood spots and buffy coats displayed considerably concordant DNA methylation profiles, their correlation scores were slightly lower than for the technical replicates. Given this, we further investigated the consistency of these discordant probes between the two groups. The F-test was performed on the full dataset which revealed a subset of 5,723 probes with q-values (adjusted p-values) of 0.01 or less (Additional file 1: Table S2). The number of Differentially Methylated Probes (DMPs) might be fractional to the full set of > 470,000 probes but it is possible that there is a small but consistent change in methylation signature due to differences in preparation methods and/or storage conditions between the two sample types. We also cannot rule out the possibility of the differences in cell population between buffy coat and blood spot samples (i.e. whole blood).

We visualised these DMPs on scatterplots and confirmed highly consistent patterns between the two groups (Additional file 1: Figure S3). These probes were located at almost identical spots on the scatterplots for different individuals. Interestingly, the majority of these probes was apparently hypermethylated for both groups but was slightly less methylated for dried blood spots. Nevertheless, the discordance in β values of these probes generally did not exceed the platform’s commonly accepted sensitivity of 0.2 β values (Additional file 1: Figure S4) [10]. These probes were neither discordant between technical replicates, nor in the same sample types between unrelated individuals (Additional file 2: Figure S5). Processing the data using other normalisation methods (e.g. ssn, quantile), transformed the data significantly but the large proportion of these probes was still present (data not shown).

It is possible that these DMPs represent technical artefacts arising from the method of sample storage. Of the 5,723 probes, 1,516 were not associated with any gene, while the rest were associated with 3,370 different genes. The greatest proportion of these DMPs was located within a gene body, not associated with a CpG island, and were Infinium Type II design probes (Additional file 1: Table S3). A proportion (1,006) of these probes also overlapped a SNP (Additional file 1: Table S2) and some probes covered more than one CpG dinucleotide. Many of these covariates on the HM450 platform can potentially influence hybridisation efficiency, thus influencing the resulting measurements, and are the topic of ongoing discussion [9,17]. Further studies are required to address such potential technical artefacts.

## Conclusion

In summary, we have demonstrated the utility of DNA extracted from archived dried blood spots for detecting genome-wide DNA methylation profiles using the Infinium HumanMethylation450 platform. Although dried blood spot DNA can degrade, especially over prolonged storage times/period [16], we were able to obtain high quality data comparable to those obtained from DNA derived from matched frozen buffy coats. We also report that the majority of probes showed highly comparable methylation patterns within matched samples, suggesting that dried blood spot samples are suitable for these types of analyses. Furthermore, although we used DNA isolated from twenty 3.2 mm dried blood spot punches in this study, this could potentially be reduced to a few punches where starting material is limited. Our findings will greatly increase the scope of biological resources suitable for Epigenome Wide Association Studies (EWAS) [18,19].

## Methods

### DNA purification from buffy coats and dried blood spots

Three year old-archived dried blood spots and matching frozen buffy coats from five individuals were selected from the Melbourne Collaborative Cohort Study biorepository [3] (Additional file 1: Table S1). At the time of collection, blood spots and buffy coat samples were simultaneously prepared. Dried blood spots were prepared in custom printed Diagnostic Cellulose filter paper (Whatman, Kent, United Kingdom) and stored in air-tight containers at room temperature and buffy coat samples were stored at -80°C as cell pellets. A total of 12 matched samples, including one technical replicate pair (same DNA hybridised onto different arrays on same slide), were selected.

DNA extractions from buffy coats were performed using Qiagen mini spin columns (Hilden, Germany) while dried blood spot DNA was extracted using a method developed in-house: Twenty 3.2 mm diameter archived blood spot punches were added to 200 ul phosphate buffered saline and homogenised using the TissueLyser (Qiagen), which enables a more complete removal of blood materials from the filter paper. The resulting supernatant was transferred to a clean 1.5 ml tube and DNA extracted using Qiagen mini spin columns according to the manufacturer’s protocol. The quality and quantity of DNA was assessed using the Quant-iT™ Picogreen® dsDNA assay measured on the Qubit® Fluorometer (Life Technologies, Grand Island, NY). All bisulfite conversion experiments (EZ DNA Methylation-Gold kit, Zymo Research, Irvine, CA), quality control analyses and the Infinium HM450 DNA methylation assays were performed at the Australian Genome Research Facility as per the manufacturers’ instructions.

### Illumina Infinium HumanMethylation450: data processing

Data were imported to an R environment and assessed using the *minfi* package version 1.2.0 (http://www.bioconductor.org) [20]. The overall data quality for individual samples was examined by calculating mean detection p-values. The raw colour channels were corrected using the internal control probes and converted into methylation levels. Subset-quantile Within Array Normalisation (SWAN) normalisation was performed to correct for technical discrepancies between Type I and Type II [21]. Probes with detection P-values above 0.01 were considered to be technical noise and excluded from further analysis. Probes on X and Y chromosomes were removed to eliminate any differences attributable to the “sex chromosomes” [9]. M-values and absolute methylation (β) values were calculated using *minfi.* Statistical tests were performed using M-values. Unsupervised clustering plots were generated using the *lumi* R package and the variable probes were selected according to coefficient of variance, which is calculated by standard deviation divided by mean across individuals [22]. DMPs were identified using an F-test in *minfi*.

### Ethics declaration

The blood samples were collected as a part of the Melbourne Collaborative Cohort Study. Written informed consent was obtained from each participant. This study was approved by the Human Research Ethics Committee of the Cancer Council Victoria (Project IEC 9001) and meets the principles of the Declaration of Helsinki.

### Availability of supporting data

All lists of probes mentioned in the manuscript are available in supplementary tables. The dataset will be available in the GEO.

### Consent

Written informed consent was obtained from all participants for the use of their data for this research and the publication of any findings in the scientific literature.

## Abbreviations

BC: Buffy Coat; GC: Guthrie Card; EWAS: Epigenome-Wide Association Study; HM27: (Infinium) Human Methylation 27 k; HM450: (Infinium) Human Methylation 450 k; DMP: Differentially Methylated Probe; IARC: International Agency for Research on Cancer; SWAN: Subset-quantile Within Array Normalisation

## Competing interests

The authors declare no conflicts of interest.

## Authors’ contributions

This study was first proposed and designed by MS, GG, JH, and DE. The analyses were performed by JJ, EW, LB, CJ and the manuscript was constructed by JJ, EW, MS. Materials used in this study were prepared by HT. DP, NC and GS put significant intellectual contributions. All authors read and reviewed the manuscript.

## Supplementary Material

Additional file 1: Table S1 Table of samples used in the study. 1 GC denotes Guthrie Card (Dried blood spot) DNA and BC denotes Buffy Coat DNA. 2 Mean Detection p-value of all probes for each sample. **Table S2.** List of DMPs between dried blood spot samples and flash frozen buffy coat samples. **Table S3.** Probes Design type and genomic location of 5,723 differentially methylated probes. **Table S4.** NanoDrop spectrophotometer 260/230 and 260/280 readings. Click here for file

Additional file 2: Figure S1 Unsupervised clustering plot on the methylation (M) values of the entire set of detected probes. Technical replicates show the greatest similarities to each other. Samples extracted from same individuals generally show close relationships. However, individual 4 and 5 exhibit slightly higher similarities within sample types than within individuals. **Figure S2.** Scatterplot matrix showing relationships in DNA methylation (Beta values) between samples and Pearson’s correlations between technical replicates (light shade), within matched samples (dark shade) and between unrelated individual samples (not shaded). The Pearson’s correlations were calculated across all detected probes. Notably higher correlations were detected within technical replicates and matched pairs than between unrelated individuals. GC – Guthrie spot (dried blood spot) samples, BC – Buffy coat samples. **Figure S3.** Scatterplots of matched dried blood spot and buffy coat βvalues of individuals 1 to 5 (A to F). The differentially methylated probes between dried blood spot and buffy coat groups were highlighted in red. A, Scatterplot of Guthrie spot and buffy coat samples from individual 1. B, Scatterplot of technical replicate Guthrie spot and buffy coat samples from individual 1. C. Samples from individual 2, D, Samples from individual 2. E, Samples from individual 4. F, Samples from individual 5. **Figure S4.** Histograms Showing Mean Differences in DNA methylation values (Δβ) with normal curves. A, Mean differences in β-values (Δβ) between matched dried blood spot and buffy coats samples (5 pairs). B, between technical replicates (2 pairs). Mean differences between two sample groups were generally less than 0.1. Figure S5 Scatterplots of technical replicates (A and B) and dried blood spots (C)/buffy coats (D) of unrelated individuals. Red dots highlight DMPs between two tissue types. Note the DMPs between sample groups are highly correlative between technical replicates (A and B), same sample types between unrelated individuals (C and D). X and y axes – β values. Click here for file
