# Supplementary figures and images for: The use of DNA from archival dried blood spots with the Infinium HumanMethylation450 array
Source: BMC Biotechnol. 2013 Mar 15;13:23. doi: 10.1186/1472-6750-13-23 (PMC3610215; doi:10.1186/1472-6750-13-23)

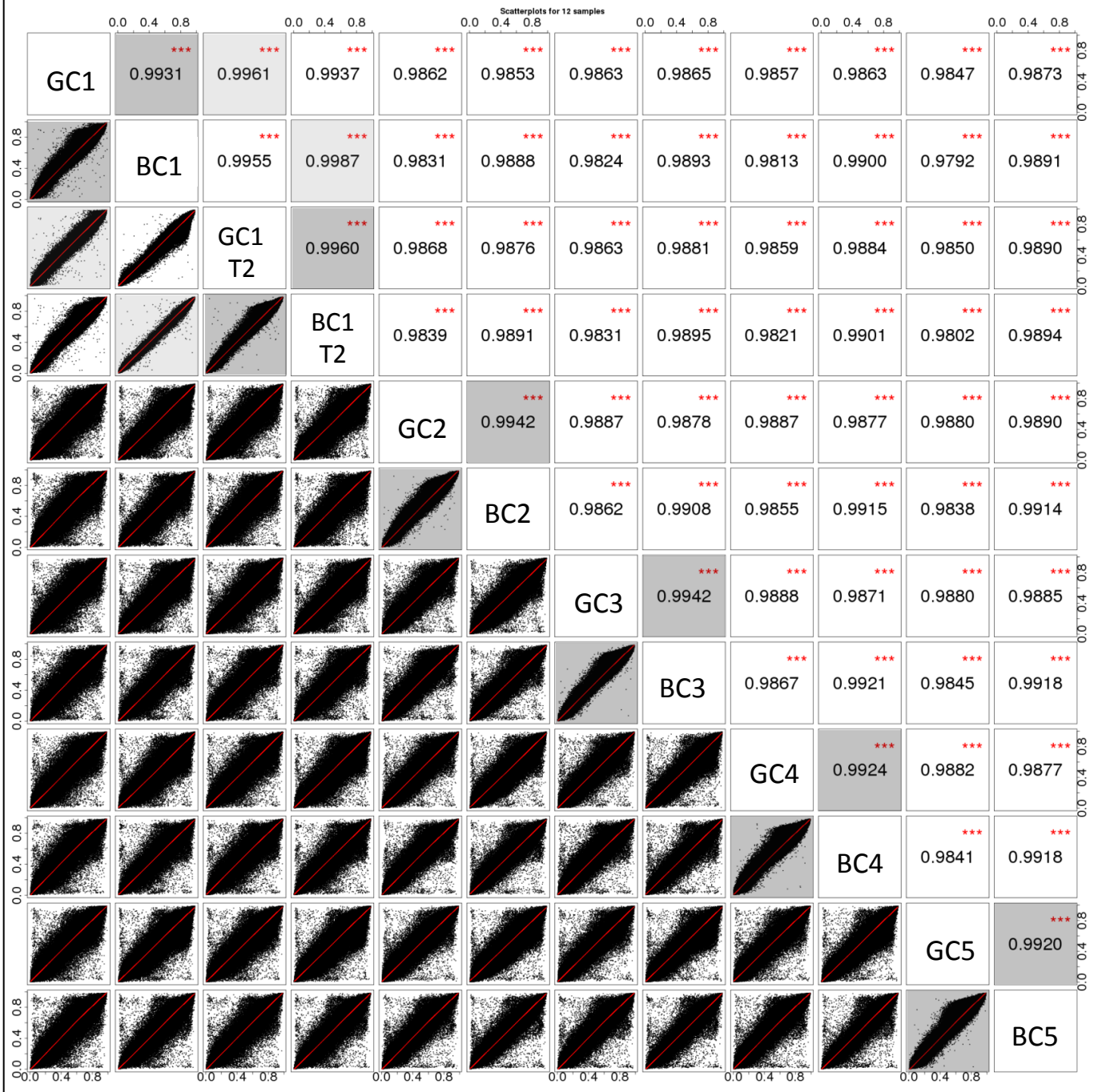

Supplementary Figure 1

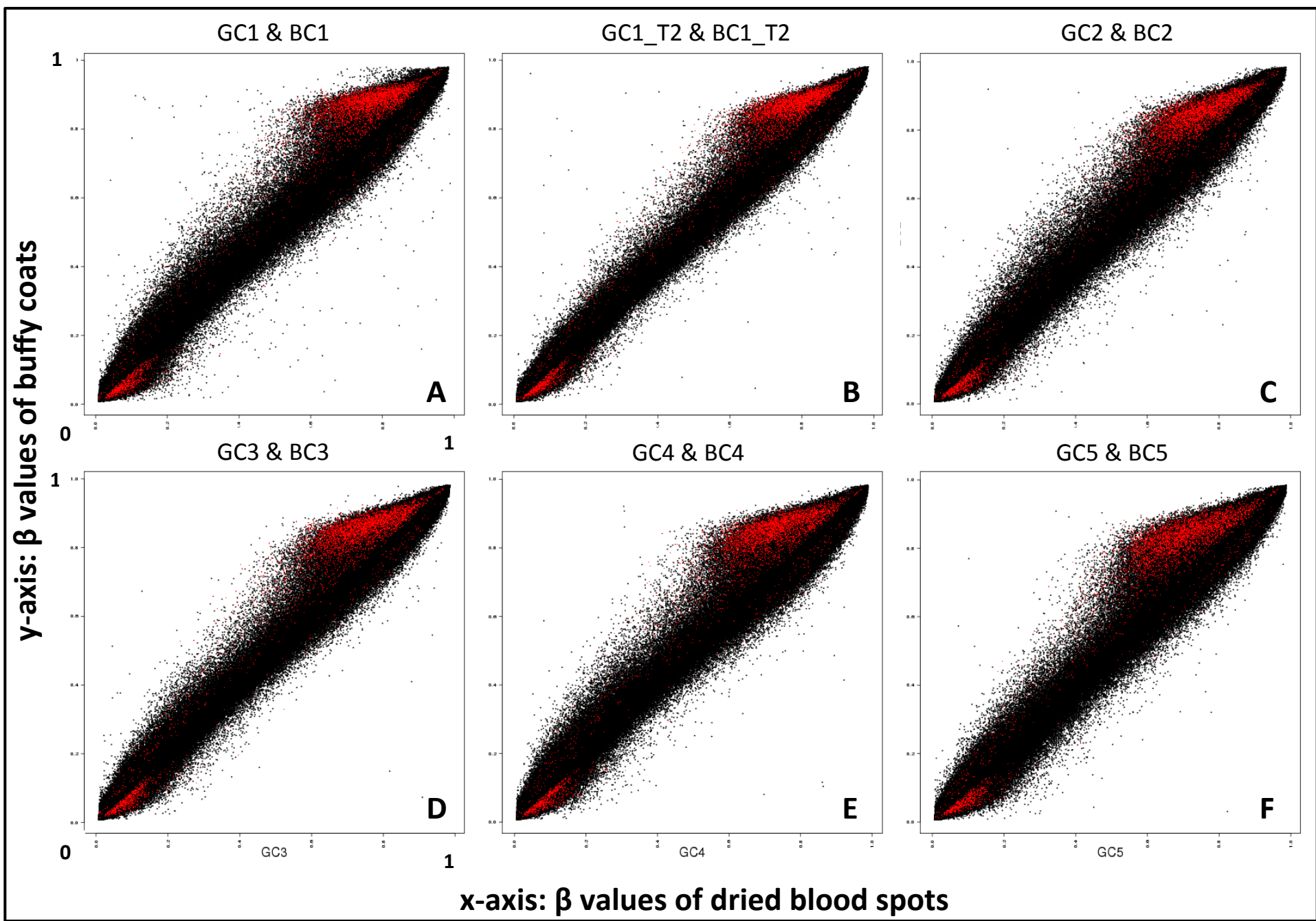

Supplementary Figure 2

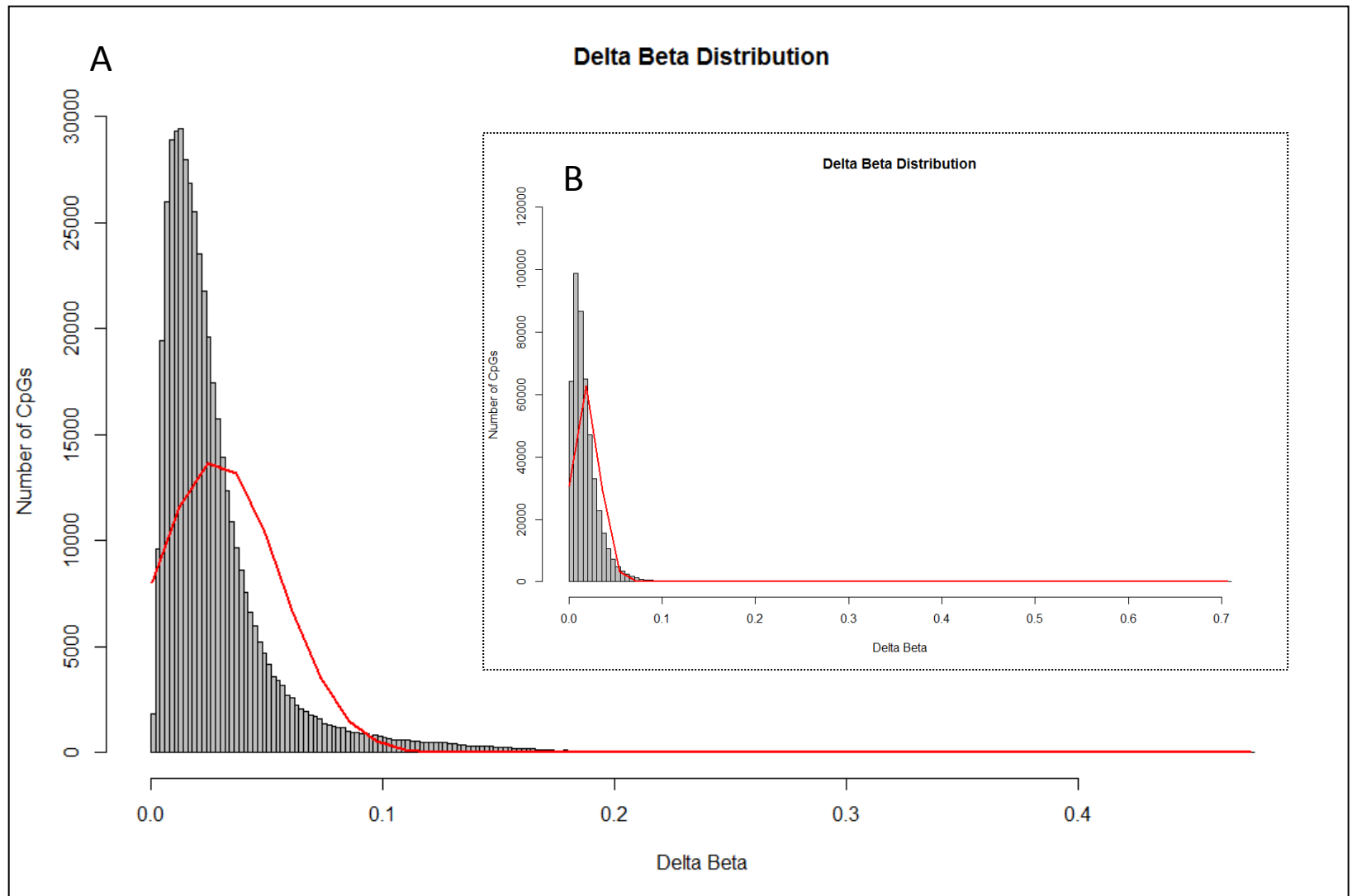

Supplementary Figure 3

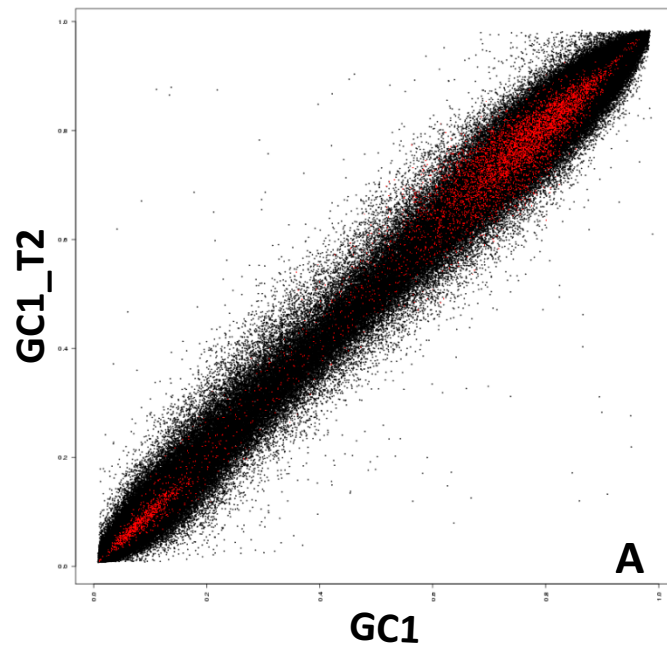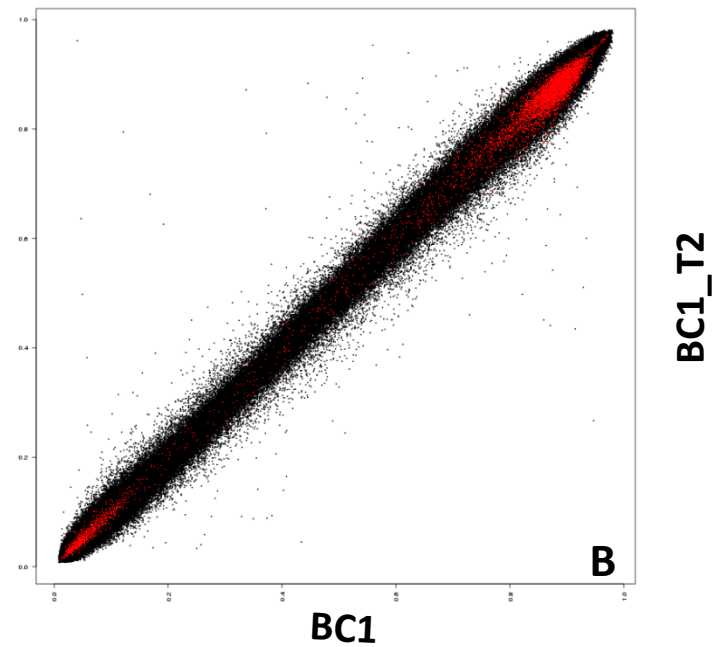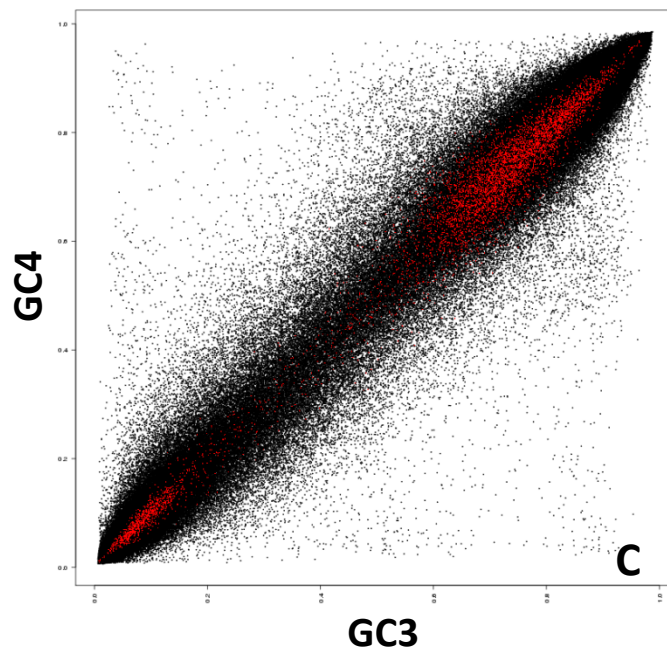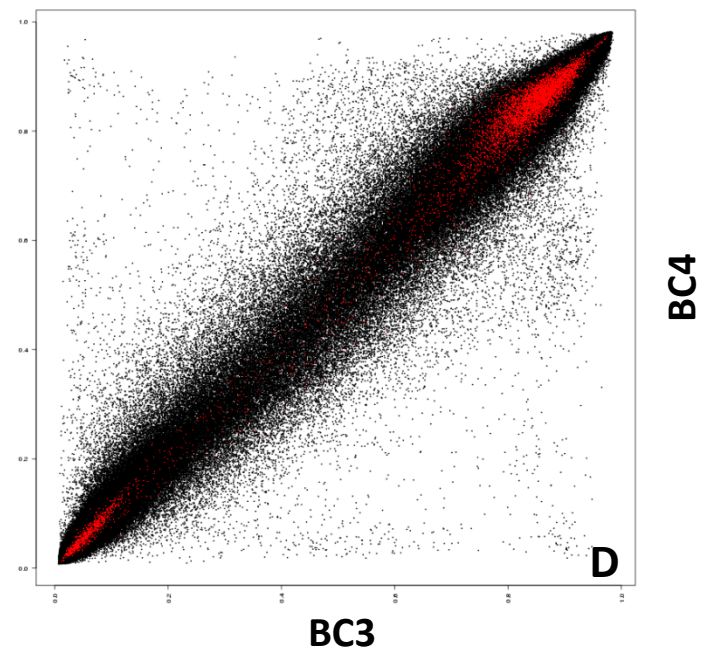

Supplementary Figure 4

Supplement: Additional file 2: Figure S1 — Unsupervised clustering plot on the methylation (M) values of the entire set of detected probes. Technical replicates show the greatest similarities to each other. Samples extracted from same individuals generally show close relationships. However, individual 4 and 5 exhibit slightly higher similarities within sample types than within individuals. Figure S2. Scatterplot matrix showing relationships in DNA methylation (Beta values) between samples and Pearson’s correlations between technical replicates (light shade), within matched samples (dark shade) and between unrelated individual samples (not shaded). The Pearson’s correlations were calculated across all detected probes. Notably higher correlations were detected within technical replicates and matched pairs than between unrelated individuals. GC – Guthrie spot (dried blood spot) samples, BC – Buffy coat samples. Figure S3. Scatterplots of matched dried blood spot and buffy coat βvalues of individuals 1 to 5 (A to F). The differentially methylated probes between dried blood spot and buffy coat groups were highlighted in red. A, Scatterplot of Guthrie spot and buffy coat samples from individual 1. B, Scatterplot of technical replicate Guthrie spot and buffy coat samples from individual 1. C. Samples from individual 2, D, Samples from individual 2. E, Samples from individual 4. F, Samples from individual 5. Figure S4. Histograms Showing Mean Differences in DNA methylation values (Δβ) with normal curves. A, Mean differences in β-values (Δβ) between matched dried blood spot and buffy coats samples (5 pairs). B, between technical replicates (2 pairs). Mean differences between two sample groups were generally less than 0.1. Figure S5 Scatterplots of technical replicates (A and B) and dried blood spots (C)/buffy coats (D) of unrelated individuals. Red dots highlight DMPs between two tissue types. Note the DMPs between sample groups are highly correlative between technical replicates (A and B), same sample types between [file 1472-6750-13-23-S2.pdf]
